# Complementary and Alternative Medicine in the Treatment of Chronic Pelvic Pain in Women: What Is the Evidence?

**DOI:** 10.1155/2013/469575

**Published:** 2013-11-28

**Authors:** Sara Paiva, Márcia Mendonça Carneiro

**Affiliations:** Department of Obstetrics and Gynecology, Universidade Federal de Minas Gerais (UFMG), Avenida Alfredo Balena 110, Santa Efigênia, 30150-270 Belo Horizonte, MG, Brazil

## Abstract

Chronic pelvic pain (CPP) is defined as pain of at least 6 months' duration that occurs in the lower abdomen or below the umbilicus and has resulted in functional or psychological disability or required intervention and treatment. Therapeutic interventions center around the treatment of CPP as a diagnosis in and of itself, and treatment of specific disorders that may be related to CPP. A multidisciplinary approach for diagnosis and treatment seems to be most effective for symptomatic relief. This paper reviews the evidence for such interventions as psychological treatments including the use of complementary and alternative medicine techniques for CPP in women. Unfortunately, finding the best evidence in this setting is difficult as only very few randomized controlled trials are available. A combination of treatments is usually required over time for the treatment of refractory CPP. The multifactorial nature of CPP needs to be discussed with the patient and a good rapport as well as a partnership needs to be developed to plan a management program with regular followup. Promotion of a multidisciplinary approach which includes complementary and alternative medicine techniques in managing CPP in women seems to yield the best results.

## 1. Introduction

### 1.1. Definition and Epidemiology

An estimated one in three people suffer from chronic pain, a condition frequently associated with decreased health-related quality of life (HRQoL) and high levels of psychological distress [1]. Despite conventional healthcare utilization, nearly half of patients with chronic pain report their pain as not under control [2]. Limitations of drug therapy for chronic pain reflect the complex pathophysiology of the condition as well as the profound contribution of psychosocial factors to the perpetuation of pain and suffering [3, 4]. 

Chronic pelvic pain (CPP) is defined as pain of at least 6 months' duration that occurs in the lower abdomen or below the umbilicus and has resulted in functional or psychological disability or required intervention and treatment. The pain may be recurring or constant [5]. Estimating the prevalence of CPP in women is challenging in part due to lack of consensus in the definition of CPP among investigators, and to the fact that only one third of women with CPP seek medical care [6–8]. Studies have quoted the range to be anywhere from 4% to 40% [6–9]. Even with a likely underestimation of prevalence, CPP accounts for 10% of indications for hysterectomy [10]. This yields 881.5 billion dollars in health care costs in the United States per year [9]. 

Although several causes of chronic pelvic pain (CPP) are recognized (Table 1), in many women a definite diagnosis remains elusive. In addition, only a few randomised controlled trials on treatment of CPP have been conducted. Given the complexity and not well-understood etiology of CPP and the possible multiple contributing etiologies, its treatment is often unsatisfactory and limited to partial symptom relief. Thus the successful treatment of women with CPP often requires a multidimensional approach [3, 5]. As a biopsychosocial understanding of chronic pain has recently become more sophisticated, a variety of psychologically based treatment approaches have been developed and empirically validated for helping people better manage their pain [1–6].

We aimed at reviewing the evidence for global interventions and, more specifically, psychological treatments as well as the use of complementary and alternative medicine techniques for the treatment of CPP in women. We performed a review of the relevant articles without language restriction based on a PUBMED search of the keywords: chronic pelvic pain, stress, endometriosis, complementary and alternative medicine, acupuncture, behavioral therapy, psychotherapy, and mind-body medicine. 

### 1.2. Causes of CPP

The consensus definition of CPP is quite broad and falls short of defining the specific causes and manifestations of CPP [8]. CPP has many potential causes and is often a complex disorder with multiple contributing etiologies (Table 1) [3]. Women may have more than one condition; in fact, women with more than one medical condition tend to have greater pain than women with one disorder [11]. The evaluation and treatment of women with CPP often requires a multidimensional approach given the context of a complex overlap of possible etiologies. In some women, no etiology is ever found [5]. 

Among women with CPP, endometriosis has an estimated prevalence of 33%, making it the most common gynecological diagnosis in this population [12]. To date, the classification and treatment of endometriosis has been based on a biomolecular conceptualization. However, the underlying mechanisms of endometriosis-associated CPP remain unclear. In recent years, there has been a growing interest in the standardized assessment of clinical outcomes in endometriosis [13]. Within the larger community of CPP patients, there is a growing recognition that biomedical variables, such as disease severity, cannot sufficiently explain levels of pain and psychological adjustment [14, 15]. In an outstanding 2010 study on pain and its psychological effects, Martin et al. speculate that persistent pain in endometriosis may be similar to other pain disorders in that psychosocial variables may play as great of a role as biomedical factors in pair report. Therefore, the assessment of coping mechanisms such as catastrophizing—a well-known modifier of chronic pain treatment outcomes—may shed light on the influence of psychosocial domains on gynecological pain report and treatment outcomes [16].

### 1.3. Stress Reactivity, Endometriosis, and CPP

A review of the literature in medicine and in the study of stress reactions outlines the interaction of biologic systems responsible for the retrograde menstrual flow, immune system disturbance, and inflammatory processes seen in endometriosis. Biofeedback and neurofeedback measures provide indirect information that suggests ways in which chronic overactivation of stress reactions may impact the interaction of autonomic nervous systems, immune system, and reproductive hormones involved in endometriosis and its associated symptoms [17]. Medical research and reproductive biology outline the impact of stress reactions on mechanisms responsible for endometriosis. Retrograde flow of menses through fallopian tubes is one of the most plausible mechanisms by which endometrial cells travel outside the uterus to settle on the bowel or ovaries or other places in the pelvic cavity [18–21]. The rise and fall of reproductive hormones over the menstrual cycle is another ingredient necessary for the proliferation of endometrial cells [21]. Women are at increased risk for both major depression and chronic pain syndromes such as CPP, and are more likely to report antecedent stressful events, have higher rates of physical and sexual abuse, and subsequently develop posttraumatic stress disorder [22]. 

Immune system disruption is also key to implantation and proliferation of this tissue outside the uterus [23–25]. The timing and interaction of these biologic reactions, over time, produce the symptoms of endometriosis, with impact on health and reproduction. There appears to be three clinically distinct forms of endometriosis: peritoneal endometriosis, ovarian endometriomas and rectovaginal endometriotic, nodule. All three types may be variants of the same pathologic process or they can be caused by different mechanisms [18–21].

Stress research documents the impact of immediate and prolonged stress reactions on the mechanisms of endometriosis development [23]. Oxidative stress which exceeds the neutralizing capacity of the natural antioxidant mechanisms may also trigger endometriosis. On the other hand, impaired defense mechanisms against OS have also been reported in women with endometriosis [24, 25].

The experience of acute stress immediately activates hypothalamic production of corticotrophin-releasing hormone (CRH), triggering a response from the sympathetic nervous system (SNS), and initiating the production of epinephrine and norepinephrine by the adrenal medulla. Acute stress responses distribute metabolic activity away from digestion and reproduction, allowing the organism to use energy for dealing with a threat; this has both direct and indirect effects on development of endometriosis symptoms [26–28]. 

The stress system suppressive effects on female reproduction involve suppression of the hypothalamic-pituitary-ovarian axis at the hypothalamic, pituitary, ovarian, and uterine levels. Experimental and human data suggest that adverse prenatal stimuli, of either maternal or fetal origin, acting in the developing embryo in utero, can lead to the development of short- and long-term health disorders. These include preterm birth of the offspring, low birth weight, and the development of adult diseases ranging from the metabolic syndrome to several neurodevelopmental disorders [28].

Elevated levels of CRH and catecholamines interact and interfere with estrogen and progesterone as well as with other biochemical regulating fallopian tube motility [29, 30]. When stress reactions coincide with menstrual flow, the potential for retrograde blood flow is present. The fall in progesterone just before menstruation activates production of prostaglandin E_2_, increasing seizure-like electrical activity of fallopian neurons. This is a likely time for fallopian tube reflux activity, which can carry endometrial cells backward away from the uterus [21]. SNS activity also produces a rapid rise in blood glucose levels, whereas parasympathetic nervous system (PNS) activity stimulates glycogen synthesis by the liver [28–32]. Glucose metabolism and insulin levels interact with prostaglandin pathways to alter immune system cells function. Catecholamine receptors on immune system cells provide another mechanism by which the immune system responds to stress reactions. Chronically elevated catecholamine levels are associated with pain and inflammatory disease, both often part of endometriosis [33–35]. Brief periods of stress reactions increase catecholamines without activating a rise in cortisol. Repeated activation of the SNS and chronically elevated levels of catecholamines generally lead to elevated cortisol levels [34]. 

Prolonged activation of CRH stimulates pituitary-derived adrenocorticotropic hormone (ACTH), which stimulates production of cortisol by the adrenal cortex. Elevated levels of cortisol circulate through the bloodstream, influencing metabolic shifts and distribution of energy throughout the brain and body. Rising levels of cortisol also inhibit CRH production and thus turn off ACTH. Under certain conditions, this regulation of stress reactions fails. Both prolonged elevation of cortisol levels and depressed cortisol levels are associated with chronic stress and can impact symptom development [35]. Although the effects of elevated cortisol are perhaps better studied, depressed cortisol levels have the potential for equally important impact on the development and expression of endometriosis symptoms [27, 36]. However, the pathways of biologic reactivity have not been well documented in women with endometriosis nor have factors associated with chronic levels of stress reactivity been distinguished from the stress of dealing with endometriosis symptoms and treatment [17].

A recent study revealed that psychosocial factors such as stress perception and quality of life are impaired in women who suffer from endometriosis. The psychoemotional alterations can be so prominent that it reaches levels of depression. No differences could be observed with regard to the social support perceived by the women, which suggests that psychoemotional alterations such as stress perception, quality of life, and depressive symptoms are not confounded by the lack of social support [37]. In 1988, Kellner et al. demonstrated that levels of depression and anxiety were significantly higher in patients with CPP and that these variables correlated with hypochondriacal beliefs. They also suggested that the psychological distress associated with pain symptoms may perpetuate the condition [38, 39]. Others argue that, in a substantial proportion of women with CPP, the cause of this distressing condition lies outside of the pelvic region and represents a functional somatic syndrome [40].

## 2. Therapeutic Interventions

As described by Fred Howard in an excellent 2003 review on CPP, therapeutic interventions center around two approaches: (1) treatment of CPP as a diagnosis in and of itself and (2) treatment of specific disorders that may be contributing to CPP [41]. These two approaches could also be described as global versus disease specific. A multidisciplinary approach is needed for diagnosis, but a multimodal approach involving both treatment approaches is often most effective for symptomatic relief. According to Howard, the global treatment of CPP can be classified into three categories: pharmaceutical, psychological, and neuroablative [41]. 

A recent Cochrane [7] review aimed to combine information from studies using many different interventions and outcome measures to provide an overview of the most effective, acceptable, and least invasive treatment options in CPP. The authors considered studies of any intervention including lifestyle, physical, medical, surgical, and psychological treatments. Outcome measures were pain rating scales, quality of life measures, economic analyses and adverse events. This review identified nineteen studies, fourteen of which were included for analysis. The authors concluded that since the pathophysiology of chronic pelvic pain is not well understood, its treatment is often unsatisfactory and limited to symptom relief. Currently, the main approaches to treatment include counseling or psychotherapy, attempts to provide reassurance using laparoscopy to exclude serious pathology, progestogen therapy such as with medroxyprogesterone acetate and surgery to interrupt nerve pathways (Table 2).

### 2.1. Psychotherapy

Psychotherapy can be incorporated as part of a multidisciplinary approach to CPP and has been demonstrated to improve management of pelvic pain and other pain syndromes, although in many studies the effect is small [16, 42, 43]. Psychotherapy can be implemented in many forms, including counseling, group therapy, cognitive behavioral therapy, and biofeedback. The use of somatocognitive therapy in the treatment of pelvic pain was evaluated in a recent randomized controlled study. When combined with standard gynecologic care, somatocognitive therapy improved psychological stress, pain experience, and motor functions of women with CPP [44]. Cognitive behavioral therapy, alone or within the context of a pain rehabilitation program, has the greatest empirical evidence for success [44–46]. Although this intervention may be limited by cost or patient compliance or acceptance, psychological treatment may decrease suffering and disability in CPP patients [41].

### 2.2. Behavioural Therapy

Through cognitive behavioral therapy, patients can develop better coping strategies and alter their pain beliefs for their chronic pain. Studies have shown that these changes can predict positive treatment outcomes by reducing helplessness, increasing perceived control, and decreasing catastrophizing [47, 48].

A 2012 Cochrane review tried to determine the effectiveness of any behavioural interventions for the treatment of primary or secondary dysmenorrhoea when compared to each other, placebo, no treatment, or conventional medical treatments. Behavioral therapies assume that psychological (the mind) and environmental factors interact with, and influence, physical processes, for example stress might influence period pain. Behavioral therapies focus on both physical and psychological coping strategies for symptoms such as pain rather than focusing on medical solutions for any underlying causes of symptoms. An example of a behavioral therapy is using relaxation to help a woman cope with painful period cramps. This review found that progressive muscle relaxation with or without imagery and relaxation may help with spasmodic (acute, cramping pain) symptoms of period pain. In addition, pain management training and relaxation plus biofeedback may help with period pain in general. The results, however, are not conclusive due to the small number of women in the trials and the poor methodological quality and age of the trials [47].

### 2.3. Mind-Body Medicine (Antistress Medicine)

The National Center for Complementary and Alternative Medicine (NCCAM) defines mind-body medicine by a range of therapies intended to enhance the mind's capacity to improve bodily function and symptoms [48]. Despite consensus that mind-body therapies can be effectively incorporated into comprehensive management of chronic pain, only 20% of chronic pain patients report using such interventions [49, 50]. A better understanding of the effectiveness of particular mind-body therapies for specific patient subpopulation may support wider and more successful integration of mind-body medicine with conventional pain management [51]. 

#### 2.3.1. Mindfulness Meditation 

Mindfulness-based stress reduction (MBSR) is a group intervention that appears to be a promising adjunct to treating chronic pain and attendant reduction in physical functioning and psychological well-being [52–54]. The core of MBSR is intensive training in mindfulness meditation and its applications for daily living and coping with stress, illness, and pain [55, 56]. Mindfulness meditation is the practice of paying attention, on purpose, moment-to-moment, in a way that is nonjudgmental and nonreactive. Practitioners report greater equanimity and less distress secondary to uncomfortable sensations, thoughts, and emotions [53, 54, 57].

A series of early treatment outcome studies found that MBSR program participants with various self-reported chronic pain conditions demonstrated significant changes in pain intensity, medical symptoms, psychological symptoms, coping ability, and inhibition of daily activity by pain, most of which were superior to standard medical care alone and persisted up to four years later [58–60]. Another descriptive study of patients with heterogeneous chronic pain condition reported significant changes in self-report measures of pain, pain beliefs, and psychological symptoms following MBSR combined with conventional medical treatment [61]. 

#### 2.3.2. Mindfulness Meditation and the Mind

Advanced studies indicate that one salutary mechanism of mindfulness appears to involve reshaping ways of thinking that engender improved emotional well-being [62–64]. Together, these studies indicate that one salutary mechanism of mindfulness appears to involve reshaping ways of thinking that engender improved emotional well-being. In addition to the mental health benefits of meditation practice and cultivating mindful awareness in daily life, simply being in a mindful state momentarily is associated with a greater sense of well-being [65]. Research further suggests that people with higher levels of mindfulness are better able to regulate their sense of well-being by virtue of greater emotional awareness, understanding, acceptance, and the ability to correct or repair unpleasant mood states [66–68]. The ability to skillfully regulate one's internal emotional experience in the present moment may translate into good mental health long-term [69].

#### 2.3.3. Mindfulness Meditation and the Body

There is increasing scientific evidence to support the therapeutic effects of mindfulness meditation training on stress-related medical conditions. In addition, research has consistently shown that mindfulness training reduces symptoms of stress and negative mood states, and increases emotional well-being and quality of life, among persons with chronic illness [54, 67, 70, 71]. The use of mindfulness training in treating specific pain conditions is presently under investigation in research supported by the National Institutes of Health [70]. The beneficial physical effects of mindfulness training may occur, in part, by learning how to better cope with the inevitable stresses of daily life, and to remember that there is usually more right with the body than wrong [69]. It has specifically been postulated that mindfulness may preempt stress-related illness through a number of psychological, biological, and behavioral pathways, including (a) clarifying primary appraisal of stressors, (b) facilitating accurate secondary appraisal of stressor demands and coping resources, (c) mitigating dysfunctional coping styles, such as catastrophizing and ruminating, (d) enhancing adaptive coping processes, such as positive reappraisal, and (e) reducing distress and psychophysiological activation [72]. Fox et al. [73] enrolled 22 women with CPP were enrolled in an 8-week mindfulness program. Twelve women completed the program and had significant improvement in daily maximum pain scores, physical function, mental health and social function.

Theoretically, patients with chronic pain may benefit from mindfulness practice through various pathways. First, both sensory and affective components of pain perception itself may be modulated through the self-regulation of attention, which can be cultivated by meditation practice [74–77]. Central nervous system pain perception pathways involving the amygdala and the anterior cingulate cortex may be inhibited or downregulated with greater levels of mindfulness [78, 79]. Second, similar to cognitive-behavioral therapies, mindfulness aims to reduce reactivity to distressing thoughts and feelings that accompany and amplify pain experience [53]. Third, mindfulness has been shown to reduce psychological symptoms, including comorbid anxiety and depression, in various patient populations [67]. Negative emotional states may amplify suffering associated with pain perception [4]. Fourth, mindfulness enhances physical self-monitoring and body awareness, possibly leading to improved body mechanics and improved self-mindfulness. Fifth, relative to traditional relaxation training, mindfulness meditation is associated with greater parasympathetic activation, which can promote deep muscle relaxation and concomitant lessening of myofascial tension and irritability that may reduce pain [69, 80]. Sixth, mindfulness may buffer against stress-related mood dysfunction and psychophysiological activation by enhancing cognitive coping process, such as positive reappraisal [81], and by strengthening emotion regulation skills, such as distress tolerance [82]. 

### 2.4. Acupuncture

Although acupuncture is widely used for chronic pain, there remains considerable controversy as to its value. Acupuncture, acupressure, and transcutaneous nerve stimulation therapy have been shown to be better than placebo in women with dysmenorrhea, but data on treatment of nonmenstrual CPP are lacking. The precise mechanisms of action of acupuncture have not been elucidated so far, but may include gate control of pain pathways, increased endogenous opioid release, and altered sympathetic tone. [83]. 

Acupuncture has been studied in endometriosis but its effectiveness for pain in endometriosis is uncertain. Twenty-four studies were identified in a Cochrane review [83] that involved acupuncture for endometriosis; however, only one trial, enrolling 67 participants, met all the inclusion criteria. Thus the evidence to support the effectiveness of acupuncture for pain in endometriosis remains limited.

A randomized controlled trial on the use of acupuncture in the treatment of CPP in men [84] has found that acupuncture was twice as effective as sham acupuncture, that more acupuncture patients had a complete resolution of symptoms, and that those patients who received acupuncture had better long-term response rates.

### 2.5. Other Treatments

Musculoskeletal system has been found to be involved in genesis and perpetuation of chronic pelvic pain (CPP) and strong evidence supports that up to 80% of women with CPP present dysfunction of the musculoskeletal system. Physical therapy, including manual therapy and myofascial release, learning specific stretching exercises, and incorporating biofeedback, offers a safe and effective form of evaluation and treatment for the patient with CPP. Properly executed pelvic floor reeducation has the potential to yield great benefit with minimal risk [1, 3]. Preliminary studies show that Thiele massage appears to be very helpful for women with CPP caused by tenderness of the levator ani muscle [84].

## 3. Conclusion 

Unfortunately, finding the best evidence as far as the treatment of CPP is concerned difficult as there are very few randomised controlled trials available.

Main limitations of reviewed studies include small sample size, absence of randomization, and lack of appropriate controls as well as follow up. In refractory CPP, a combination of treatments is usually required over time. The multifactorial nature of CPP needs to be discussed with the patient and a good rapport as well as a partnership needs to be developed to plan a management program with regular followup. Promotion of a multidisciplinary approach which includes complementary and alternative medicine techniques in managing CPP in women seems to yield the best results. Although very few of these approaches have been evaluated in formal clinical trials so far, they have much to offer to those with chronic pain in terms of enhancing quality of life and pain-related coping, as well as reducing disability and pain-related interference with functioning.

## Figures and Tables

**Table 1:** Etiologies of chronic pelvic pain [3].

Gynecological	Physiologic
	(i) Primary dysmenorrheal
	(ii) Mittelschmerz
	Pathologic
	(i) Endometriosis
	(ii) Adenomyosis
	(iii) Uterine leiomyomata
	(iv) Cervical stenosis or obstructive abnormality
	(v) Pelvic venous congestion syndrome
	(vi) Ovarian remnant/residual ovary syndrome
	(vii) Pelvic adhesions
	(viii) Postpelvic inflammatory disease
	(ix) Endosalpingiosis

Gastrointestinal tract	(i) Irritable bowel syndrome
	(ii) Inflammatory bowel disease
	(iii) Chronic constipation

Urinary tract	(i) Interrstitial cystitis

Nervous system	(i) Pudendal neuralgia
	(ii) Provoked vestibulodynia

Muskuloskeletal	(i) Pelvic floor mayalgia
	(ii) Myofascial pain

Psychosocial	(i) Depression
	(ii) Physical/sexual abuse
	(iii) Drug seeking behaviour

**Table 2:** Alternative medicine therapies for the treatment of chronic pelvic pain (CPP).

Mind-body therapy	
(i) Psychotherapy	
(ii) Acupuncture	
(iii) Meditation	
(iv) Behavioral therapy	
(v) Relaxation techniques	

Pelvic floor therapy	
(i) Biofeedback	
(ii) Global therapy massage	
(iii) Myofascial massage	
(iv) Thiele massage	
